# etymologia: ***Merkel* [mәr′-kәl] *Cells***

**DOI:** 10.3201/eid1409.099999

**Published:** 2008-09

**Keywords:** etymologia, etymology, Merkel cell, Merkel cell carcinoma, Friedrich Merkel

Specialized cells found near the dermal-epidermal junction, characterized by numerous membrane-bound granules with dense cores. The cells were named after German anatomy professor Friedrich Sigmund Merkel, who experimented with osmium tetroxide staining and described these cells in 1875. First identified in the skin of a mole, they were later found in human skin. The cells are responsible for the highly malignant skin tumor known as Merkel cell carcinoma. An infectious cause for Merkel cell carcinoma has been proposed.

